# ABPEPserver: a web application for documentation and analysis of substitutants

**DOI:** 10.1186/s12885-023-10970-8

**Published:** 2023-06-03

**Authors:** Abhijeet Pataskar, Jasmine Montenegro Navarro, Reuven Agami

**Affiliations:** 1 Division of Oncogenomics, Oncode Institute, The Netherlands Cancer Institute, Plesmanlaan 121, 1066CX Amsterdam, the Netherlands; 2 Erasmus MC, Department of Genetics, Rotterdam University, Rotterdam, the Netherlands

**Keywords:** Substitutants, Codon reassignment, Translation, Cancer, Immunotherapy

## Abstract

**Background:**

Cancer immunotherapy is implemented by identifying antigens that are presented on the cell surface of cancer cells and illicit T-cell response (Schumacher and Schreiber, Science 348:69–74, 2015; Waldman et al., Nat Rev Immunol 20:651–668, 2020; Zhang et al., Front Immunol 12:672,356, 2021b). Classical candidates of such antigens are the peptides resulting from genetic alterations and are named “neoantigen" (Schumacher and Schreiber, Science 348:69–74, 2015). Neoantigens have been widely catalogued across several human cancer types (Tan et al., Database (Oxford) 2020;2020b; Vigneron et al., Cancer Immun 13:15, 2013; Yi et al., iScience 24:103,107, 2021; Zhang et al., BMC Bioinformatics 22:40, 2021a). Recently, a new class of inducible antigens has been identified, namely Substitutants, that are produced as a result of aberrant protein translation (Pataskar et al., Nature 603:721–727, 2022).

**Main:**

Catalogues of Substitutant expression across human cancer types, their specificity and association to gene expression signatures remain elusive for the scientific community's access. As a solution, we present ABPEPserver, an online database and analytical platform that can visualize a large-scale tumour proteomics analysis of Substitutant expression across eight tumour types sourced from the CPTAC database (Edwards et al., J Proteome Res 14:2707–2713, 2015). Functionally, ABPEPserver offers the analysis of gene-association signatures of Substitutant peptides, a comparison of enrichment between tumour and tumour-adjacent normal tissues, and a list of peptides that serve as candidates for immunotherapy design. ABPEPserver will significantly enhance the exploration of aberrant protein production in human cancer, as exemplified in a case study.

**Conclusion:**

ABPEPserver is designed on an R SHINY platform to catalogue Substitutant peptides in human cancer. The application is available at https://rhpc.nki.nl/sites/shiny/ABPEP/. The code is available under GNU General public license from GitHub (https://github.com/jasminesmn/ABPEPserver).

**Supplementary Information:**

The online version contains supplementary material available at 10.1186/s12885-023-10970-8.

## Background

Cancer immunotherapy has improved cancer patients' treatment possibilities, especially those with metastatic spread [19]. Although the success, the clinical outcome of immunotherapy is not consistent both within and across cancer entities. A sufficient infiltration into the tumour microenvironment and activation of effector T-cells against cancer cells can be seen as predictors for responses to T-cell-based immunotherapies [15–18, 21–23]. However, cancer cells have developed multiple mechanisms to inhibit anticancer immunity's activity, effecting antigen presentation on Human Leukocyte Antigen (HLA) class I receptor molecules [10].

**Fig. 1:**
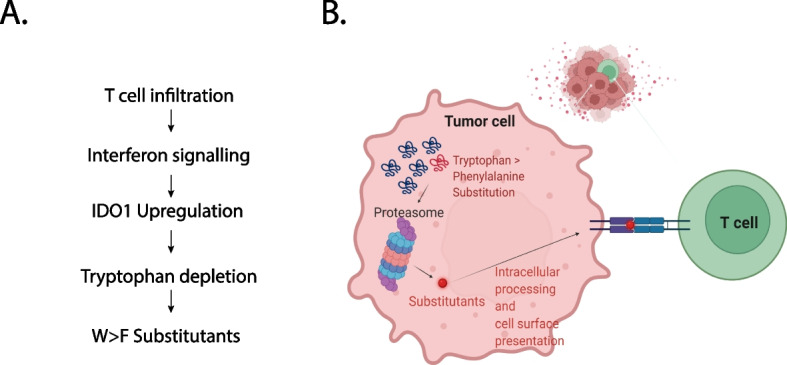
Substitutants as novel cancer antigens. **A** A flowchart depicting the pathways in cancer cells leading to the generation of W > F substitutants. **B** A model describing the mechanisms leading to the presentation of substitutant antigens and consequential T cell killing of cancer cells

Based on the responsiveness to immunotherapy, a tumour type could be classified as “hot”, i.e. responsive and “cold”, i.e. non-responsive tumours [2]. A critical delineator between these tumour-types is the identification of presentable antigens that harbour the ability to elicit T-cell responses [4]. Recently, a new class of antigens were identified, namely Substitutants, which are produced as a result of aberrant translation consequential of T cell infiltration associated endogenous tryptophan depletion [13] (Fig. 1A). Substitutants are defined as peptides arising because of Tryptophan to Phenylalanine substitution in cancer cells as a result of tryptophan shortage. Importantly, Substitutant peptides harbour immunotherapeutic potential owing to their ability to elicit T cell responses [13] (Fig. 1B). Hence, it is not only necessary to correctly identify and catalogue presentable substitutants across multiple human tumour-types, but it is also essential that we understand molecular pathways that enrich their production and presentation.

**Fig. 2:**
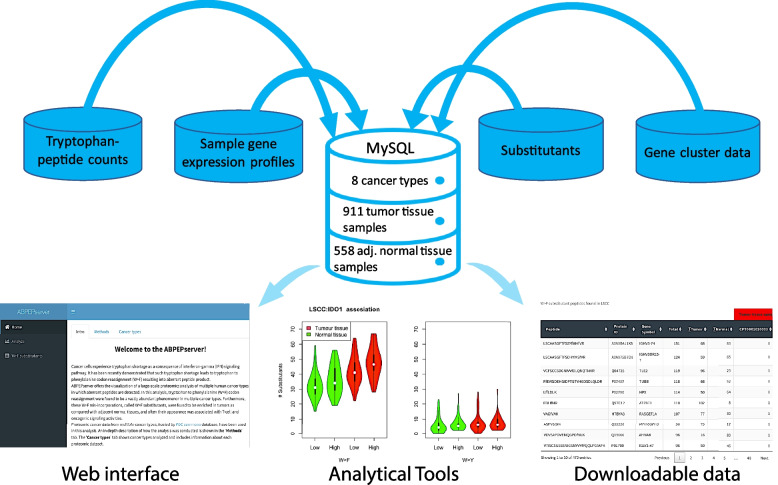
Design of ABPEPserver. A model describes the ABPEPserver. The server offers a web interface for easy data accessibility, analytical tools for biological interpretation, and the possibility of downloading relevant data in text format. The database was designed in MySQL, uses W-peptide counts, Gene expression profiles downloaded from the PDC commons Databases, identified substitutant peptides and Gene cluster data information for eight cancer types, sourcing data from 911 tumours and 558 tumour-adjacent normal tissue

To identify and characterize such substitutant peptides, we undertook a large-scale proteomic analysis of multiple cancer types sourced from the CPTAC consortium [8]. To make this analysis more accessible to the scientific community, we developed an online database called ABPEPserver, which harbours substitutant information from eight independent human tumour types, namely Lung Squamous Cell Carcinoma (LSCC, [14]), Clear Cell Renal Cell Carcinoma (CCRCC, [5]), Glioblastoma (GBM, [20]), Head and Neck Squamous Cell Carcinoma (HNSCC, [11]), Hepatocellular Carcinoma (HCC, [9]), Ovarian Sereous Cystadenocarcinoma (OVSCC, [12]), Pancreatic Ductal Carcinoma (PDA, [3]), Uterine Corpus Endometrial Carcinoma (UCEC, [7]). ABPEPserver provides background information on the substitutant peptides and their analyzed cancer type description, a comparative analysis of substitutant expression in tumours and tumour-adjacent normal tissues, and downloadable links of text files harbouring this information. These substitutant peptides can then be used for predicting neoepitopes for potential immunotherapy tests. Altogether, ABPEPserver is an ease-of-access web tool useful towards designing immunotherapeutic substitutant candidates (Fig. 2).

## Construction and content

### Proteomic analysis of substitutant peptide expression

As previously published, a reanalysis of substituants peptide expression was undertaken [13]. Briefly, Philosopher pipeline was used to detect endogenous substituants in the large-scale proteomics dataset with the same parameters as previously published [6].

Mass spectrometry-derived processed spectra (mzML) files for all independent cancer types were obtained from the CPTAC database [8]. The database of protein sequences was prepared in one of two ways. First, the human proteome with all instances of tryptophan amino acids in the proteome changed to all other amino acids except Arginine and Lysine was used as a database in the scan – referred to as database 1(fully substitutant). Second, to optimize true positives, we generated a second database (optimized database) which includes the canonical human proteome (UniPROT) with the substitutant tryptic peptides (length > 5 & < 50 amino acids) spanning tryptophan residue and tryptophan substituted to all other amino acids. The analysis of both these databases is presented separately on the server. Additional details on the FASTA file are available on the GITHUB page and the description section on the server. Briefly, MSFragger searches the mzML spectral files against the custom database for peptide detection with the following parameters; Precursor mass lower: − 20 ppm, Precursor mass upper: 20 ppm, precursor mass tolerance: 20 ppm, calibrate mass: True, Deisotoping: True, mass offset: False, isotope error: Standard, digestion: Strictly tryptic (Max. missed cleavage: 2), Variable modifications: 15.99490 M 3, 42.01060 [^ 1, 144.1021 n^ 1, 144.1021 S 1, Min Length: 7, Max Length: 50, digest mass range: 500:5000 Daltons, Max Charge: 2, remove precursor range: − 1.5, 1.5, topN peaks: 300, minimum peaks: 15, precursor range: 1:6, add Cysteine: 57.021464, add Lysine: 229.162932, among other basic parameters (Supplementary Table 1). Next, PeptideProphet validates detected peptides with the following parameters; accmass: TRUE, decoyprobs: TRUE, expectScore: TRUE, Glycosylation: FALSE, ICAT: FALSE, masswidth: 5, minimum probability after first pass of a peptide: 0.9, minimum number of NTT in a peptide: 2, among other parameters (Supplementary Table 1). Isobaric quantification was then undertaken the following parameters (bestPSM: TRUE, level: 2, minProb 0.7, ion purity cut-off: 0.5, tolerance: 20 ppm, among other parameters (Supplementary Table 1). Next, to only retain confident peptides, peptides were filtered using stringent False Discovery Rate (FDR) filtering. The following parameters were used for FDR filtering; FDR < 0.01, peptideProbability: 0.7, among other parameters (Supplementary Table 1). Next, TMT-integrator was used to create integrated reports with isobaric quantification across all samples with the following parameters (retention time normalization: False, minimum peptide probability on top of FDR filtering: 0.9, among other parameters (Supplementary Table 1).

Substitutant peptides were fetched from the reports of TMT Integrator (version 3.1.0). Using a R-script, peptides with a log2-transformed intensity score above 0 in a sample were observed as positively detected peptides in that sample. As described before [13], for intra-tumour type analysis a filter for the maximum number of samples was applied to retain peptides with higher specificity in expression, except for W > F substitutants due to their exclusive significant and specific distribution wherever significant. All tumour types have been demonstrated to be exclusive with the analysis of database 1 [13], while GBM, UCEC, and PDA did not show this exclusivity in the analysis of database 2. This optimizes the signal for gene expression correlation analysis. Furthermore, this script was used to plot bar plots depicting the cumulative number of tryptophan substitutants detected in the scans.

Gene expression data was downloaded in GCT format from PDC database. The counts of W-substitutants were combined for each sample with gene expression profiles. PERL scripts were designed to count the number of substitutants when a gene is lowly expressed (intensity < 0) or highly expressed (intensity > 0). *P*-values for comparison are calculated using Wilcoxen t-test.

### Data sources

Eight independent human tumour-types, namely Lung Squamous Cell Carcinoma (LSCC, [14]), Clear Cell Renal Cell Carcinoma (CCRCC, [5]), Glioblastoma (GBM, [20]), Head and Neck Squamous Cell Carcinoma (HNSCC, [11]), Hepatocellular Carcinoma (HCC, [9]), Ovarian Serous Cystadenocarcinoma (OVSCC, [12]), Pancreatic Ductal Carcinoma (PDA, [3]), Uterine Corpus Endometrial Carcinoma (UCEC, [7]), were analysed to generate ABPEPserver database (Table 1) [1]. CPTAC IDS of the datasets are provided in Table1 and Supplementary Table 2.

**Table 1:** This table details the tumour types used to build ABPEPserver, with the information on identified substitutants and the number of tumours and adjacent normal tissues used to build the analysis

Cancer type	PDC study ID	Substitutants	# Tumor samples	# Normal samples
Clear Cell Renal Carcinoma	PDC000127	3623	110	84
Glioblastoma	PDC000204	3978	100	10
Head and neck Squamous Cell Carcinoma	PDC000221	3212	105	53
Hepatocellular Carcinoma	PDC000198	9787	165	165
Lung Squamous Cell Carcinoma	PDC000234	8237	110	101
Ovarian Serous Cystadenocarcinoma	PDC000110	892	84	22
Pancreatic Ductal Adenocarcinoma	PDC000270	3440	137	74
Uterine Corpus Endometrial Carcinoma	PDC000125	4329	100	49

### Design of database and web tools

A MySQL database was created to efficiently organize output data and avoid storage difficulties of multiple large files. With a database, data is efficiently stored and easily retrievable. For each cancer type, we stored substitutant counts. We identified individual substitutant peptides and gene cluster data from the proteomic analysis and associated gene expression. Data for tumour and adjacent normal tissue samples are made distinguishable for comparison.

### Implementation

ABPEPserver is a R/Shiny application which allows users to interact with and visualize our data and analysis. We implemented the R package RMySQL 0.10.23 to connect our database to the application.

Users are provided with background scientific information, methods and cancer types used in the study on the home page of the ABPEPserver. Users are provided then two options for using the web tools, viz. Analyze and tryptophan to phenylalanine (W > F) Substitutants. In the “Analyze” module, the user can explore the enrichment of substitutants and their association with molecular gene expression signatures. On the other hand, the “W > F Substitutants” module can be employed to browse individual substitutants in multiple cancer types and tumour vs adjacent normal tissue expression. Here, the information on the database used to detect the peptide is also added. The corresponding files of this module are downloadable.

## Utility and discussion

### Description of utility

ABPEPserver is a web database that serves as a platform to identify and characterize the expression of substitutants, a recently identified class of aberrant proteins with immunotherapeutic potential, across various human cancer types. In addition, ABPEPserver allows the analysis of the association of molecular gene expression signatures to the enrichment of substituants. As an example, such analysis was demonstrated to be essential for pinpointing the role of T-cell infiltration and direct causal proteins (such as IDO1) in the expression of W > F substitutant peptides (Fig. 2) [13]. This shows that the expression of substitutant peptides is regulated by IDO1 expression, which is induced via the T-cell infiltration pathway.

Furthermore, ABPEPserver displays enrichment differences of substitutant peptides in tumours and tumour-adjacent normal tissues, an analysis that can be utilized for underpinning cancer-specific underlying mechanisms. Lastly, downloadable text files from the ABPEPserver can be used to identify common cancer-specific substitutants that can provide the foundation for predicting neoepitopes for a wide-ranging immunotherapeutic application. Altogether, ABPEPserver provides detailed information on the identity of the substitutants, their cancer-specific expression and immunotherapeutic potential.

### User interface

#### Main page

The main page of ABPEPserver displays relevant information, methods in detail and cancer types used in the analysis in the construction of ABPEPserver along with supplementary information on each cancer type (Supplementary Fig. 1A).

#### Analyze module

The “Analyze” module allows the user to select the cancer type and database of interest and plot Barplots, scatter-contour plots and Violin plots for analytical purposes (Supplementary Fig. 1B). Barplots allow the display of various types of W-substitutants and their relative enrichment concerning each other. Scatter contour plots allow the association analysis of all proteins to the number of substitutants. Violin plots allow the analysis of individual protein association with the number of substitutants. For example, IDO1 expression was associated with substitutant peptide expression using this analysis (Fig. 2). IDO1 is an enzyme that catabolizes Tryptophan molecules in the cell; hence, the association of IDO1 expression with substitutants is biologically meaningful. Hence, Analyze module provide critical biological insights into the substitutant peptide expression and can be used by the users to design cancer-specific immunotherapy study.

#### W > F substitutants module

This module allows users to select the cancer type and database of interest and plot and download individual peptides for potential immunotherapeutic applications (Supplementary Fig. 1C). The utility of this module is demonstrated in a case study below.

### Case study: identification of immunocompetent substitutants using ABPEPserver

**Fig. 3:**
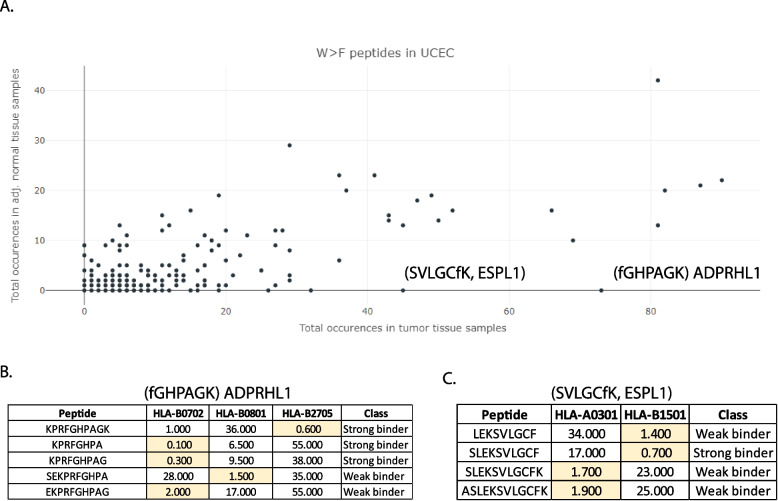
Case-study: Utility of ABPEPserver. **A** A scatter plot from ABPEPserver (W > F Substitutants module) for UCEC (Uterine Cancer), depicting total occurrences in tumour tissue samples on X-axis and Total occurrences in adj. normal tissue samples on Y-axis. Identification of two peptides that are selected to be highly tumour-specifically expressed. The database used for this scatter plot is the fully substitutant database (database 1). **B**, **C** Tables displaying peptide combinations for the selected peptides in (A) that have HLA-binding ability to one or more HLA super alleles. Rank is displayed as inverse rank from NETMHC ^23^analysis where peptides rank < 1 are identified as strong binders and rank > 1 & rank < 2 are identified as weak binders

Using the “W > F Substitutants” module for UCEC (Uterine Cancer) and the displayed scatter-plot, two example peptides ( fGHPAGK and SVLGCfK) were identified using fully substitutant database (database 1) and found to be expressed in a highly tumour-specific manner (73 tumours and 0 tumour-adjacent normal tissue, 45 tumours and 0 tumour-adjacent normal tissue respectively) (Fig. 3A). This tumour-specific expression implies that it is feasible to target these antigens specifically in cancerous tissues without harbouring any reactivity against normal cells if these peptides can present on the cell surface and bind to HLA molecules. Indeed, NETMHC [1] based prediction shows that many combinations of these two peptides have potentially strong binding affinity to one or multiple HLA super-alleles (Fig. 3B-C). This analysis indicates that the discovered peptides potentially harbour strong immunotherapeutic potential and warrant experimental validation. Thus, ABPEPserver can be used to identify potential cancer-specific antigens for immunotherapeutic applications.

### Future outlook

In future, we plan to expand ABPEPserver functionality towards harbouring other kinds of aberrant peptides that are discovered, such as ribosomal-frameshift-associated chimeric peptides. In response to tryptophan shortage, it has been observed that ribosomes change the frame at the tryptophan-associated “TGG” codon, leading to the synthesis of W-chimera. Since W-chimera were only observed in cell-culture systems, it is important to analyze whether W-chimeras is also expressed in the eight cancer types analyzed here. If the expression is observed, the next pursuit is the association of gene expression pathways.

## Conclusions

We present ABPEPserver, a database of aberrant Substitutant peptides in human cancer. Substitutant peptides result from tryptophan to phenylalanine misincorporation events and are generated in human cancer due to T-cell infiltration and subsequent tryptophan depletion [13]. The “Analyze” module of ABPEPserver allows exploration of gene expression signature of substitutant peptide expression in multiple human cancer types, organized as peptides detected in tumours and tumour-adjacent normal tissue. The W > F “Substitutant” modules allow exploration of individual Substitutant peptides in multiple patient samples and have download features. The presented case study exemplifies that the substitutant peptides identified by ABPEPserver harbour immunotherapeutic potential. Hence, ABPEPserver is a valuable resource to the scientific community invested in anti-tumour immunotherapy development.

## Supplementary Information

**Additional file 1: Supplementary Figure 1.** Description: ABPEPserver design and modules. (A) Introduction Main page of ABPEPserver provides background information, methods and Cancer-types used for the development of the server. (B) User entry page of “Analyze module” wherein user can select cancer-type of interest and one of the three Analyze sub-modules (C) User entry page “W>F Substitutant” peptides with browse function.

**Additional file 2: Supplementary Table 1.** Philosopher parameters used for scanning and detecting substitutant peptides in cancer proteomes.

**Additional file 3: Supplementary Table 2.** The table detailing publications associated to the analyzed data.

## Data Availability

The application, data, and material are freely available at https://rhpc.nki.nl/sites/shiny/ABPEP/. The code is available under GNU General public license from GitHub ( https://github.com/jasminesmn/ABPEPserver).
