# Supplementary material for: ABPEPserver: a web application for documentation and analysis of substitutants
Source: BMC Cancer. 2023 Jun 3;23:502. doi: 10.1186/s12885-023-10970-8 (PMC10239192; doi:10.1186/s12885-023-10970-8)

Supplementarty Figure 1

A.

Entry Page

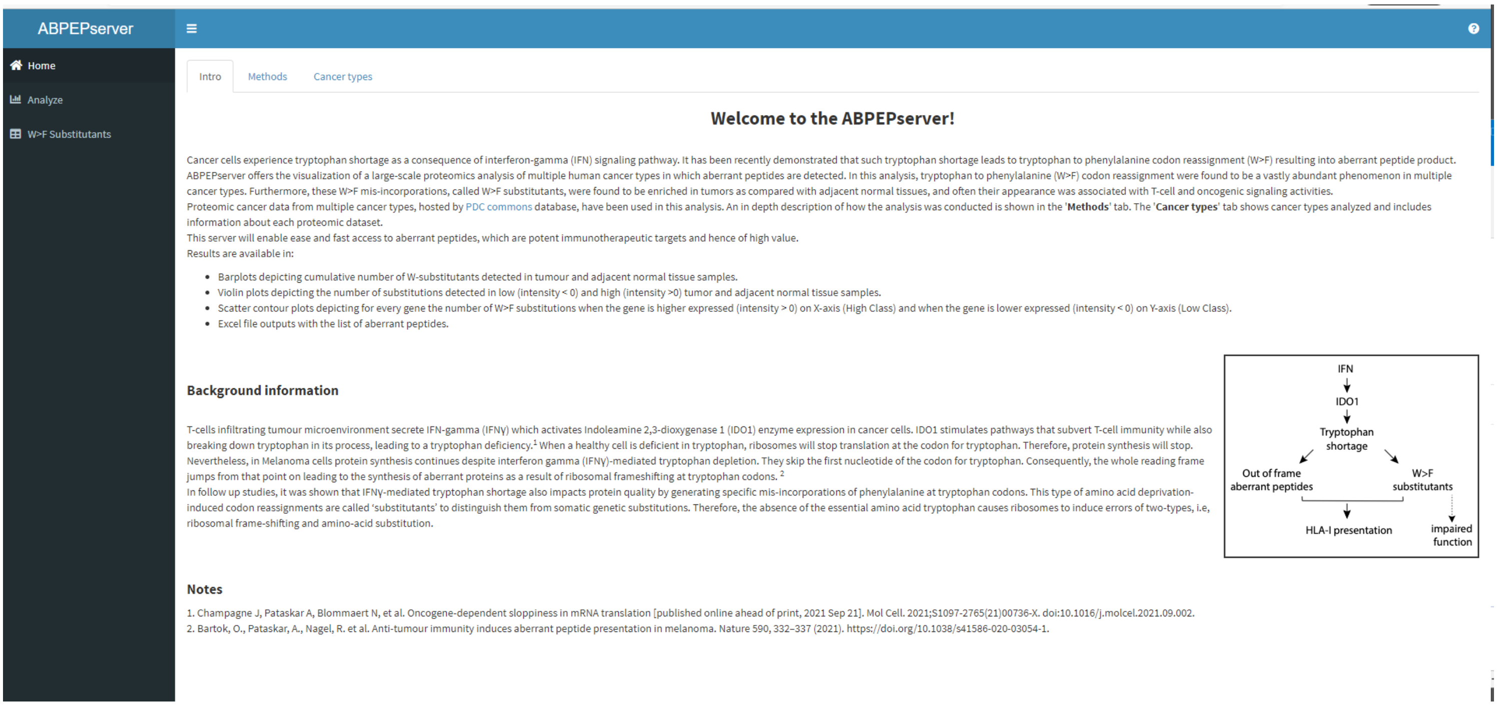

B.

Analyze Module

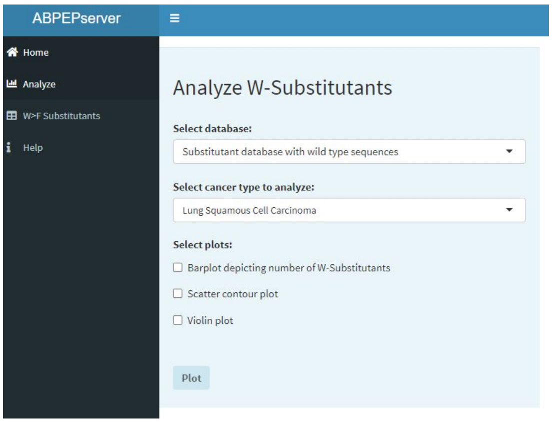

C.

Browse Peptides

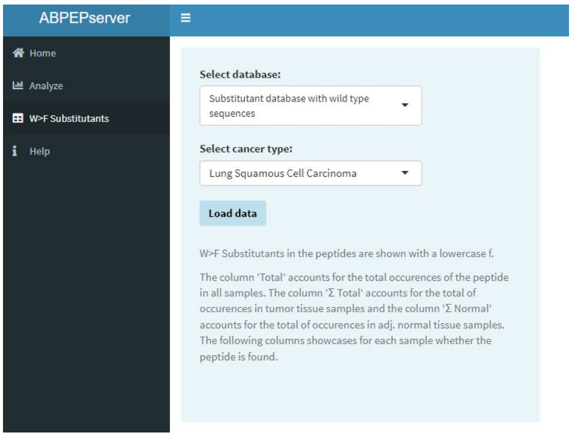

Supplement: Supplementary file 1 — Additional file 1: Supplementary Figure 1. Description: ABPEPserver design and modules. (A) Introduction Main page of ABPEPserver provides background information, methods and Cancer-types used for the development of the server. (B) User entry page of “Analyze module” wherein user can select cancer-type of interest and one of the three Analyze sub-modules (C) User entry page “W>F Substitutant” peptides with browse function. [file 12885_2023_10970_MOESM1_ESM.pdf]
